# The Madstone

**Published:** 1901-06

**Authors:** 


### THE MADSTONE.

THE veneer of civilization is very thinly laid and very little force is required to shatter it and disclose the crude savagery that lies beneath it. One of the notable relics of barbarism—of which there are many—which remain to stultify humanity and bring discouragement to the enlightened is the so-called madstone. There are very few communities which do not possess one or more to be brought into requisition when any one is bit by a rabid or supposedly rabid animal. To what geological formation the magic stone belongs is a matter of indifference, but in the popular mind there is no doubt but that certain stones when applied to the seat of injury exercise a specific effect upon the elimination of the poison. There is no question about the truth of this, because the owners of the madstones all say so. The following case which occurred in Atlanta is overwhelmingly confirmatory:

### MADSTONE STICKS TO WOUND IN LEG.

ADHERED FOR HOURS TO DOG'S BITE IN ROSWELL ISON'S FLESH—DREW OUT GREEN POISON.

Boy Was Bitten Last Monday—His Father Advertised in The Constitution Thursday and Secured a Madstone that Did Work.

Roswell Ison, thirteen-year-old son of W. L. Ison, of 350 Linden street, was bitten by a supposed rabid dog last Monday, and yesterday a madstone was applied to the wound with astonishing results.

The madstone remained on the wound for ten hours or more, not having dropped off up to a late hour last night. Apparently it drew a large quantity of poison from the wound, which was somewhat ulcerated, and long before it fell off the skin around the wound had practically resumed its normal color.

Mr. Ison secured the madstone through an advertisement in *The Constitution*. Thursday morning he advertised for such a stone, and almost immediately received three answers—one from Decatur and two from Atlanta. Two of the madstones he tried without effect, one of them being that from Decatur. The third one he secured from Mrs. Dove, of No. 184 Edgewood avenue, and it was with that the astonishing results were produced yesterday.

Roswell Ison was bitten on the right leg by a small black and white spotted dog near the corner of Peachtree and Ellis streets Monday afternoon. The dog ran on, passing a boy and a girl without disturbing them. It ran across the street and bit a negro boy. The next day Roswell heard that a messenger boy had been bitten by a dog similar to the one that bit him.

WENT TO PASTEUR INSTITUTE.

The wound on the boy's leg became irritated and began to swell. Fearing that the dog had hydrophobia, Mr. Ison had him taken to the Pasteur Institute on Auburn avenue. There he stated the circumstances to Dr. James

N. Brawner, who said that it would be impossible to determine whether the dog had hydrophobia unless the animal could be examined and experimented with. The wound was a slight one, on the lower part of the leg, and Dr. Brawner stated that only about 25 per cent. of those bitten by rabid dogs at such a place were ever infected with the disease. He said the boy could be taken for treatment if it were desired, but he hardly considered it necessary.

Mr. Ison was not satisfied and he immediately took steps to secure a madstone. He tried two of these, as stated, without effect, and the third was tried yesterday. Mrs. Dove does not own the stone, but it belongs to one of her relatives in Augusta. She did not care to have it go out of her possession, and young Roswell Ison was taken to her home for treatment.

The stone, which is small in size—about as large as the end of a man's finger, and of a reddish brown color—was first boiled in sweet milk. The wound was slightly abraded and the stone applied to it. It stuck fast as soon as brought into contact with the flesh, and soon it was filled through about half its thickness with a greenish substance, and dropped off. The portion of the stone in which the poison had accumulated turned a dark green, and it was necessary again to boil it in sweet milk, a process which removes the poison.

REMAINED ON SEVEN HOURS.

The second time it was applied to the wound it remained fast nearly seven hours before dropping off. It was again treated and applied for the third time, remaining fast to the flesh up to a late hour last night. Mr. Ison accounts for this by the fact that on the first application the stone became quickly saturated with the poison, and could contain no more. The second time it was applied there was far less poison in the wound, and it remained much longer, and the same was true of the third application. It will be kept on the wound until it will no longer adhere. Mr. Ison says that when he took hold of the stone on the boy's leg it adhered viciously, wrinkling the flesh when an effort was made to pull it away.

After the second application of the stone the swelling in the leg disappeared, and the skin resumed almost its normal color. The boy stood it well, and Mr. Ison feels now that his recovery from the effects of the bite is assured. He is a healthy boy, and so far has suffered no ill effects from the wound made by the dog.

The vicious adhesion of the stone to the wound showed an intelligent purpose and a beneficent design that would set all skepticism at nought. The demonstration was absolutely convincing and joy once more reigns in the Ison household.

The only flaws in this smooth story—trifling ones, to be sure—are that the dog was not known to be rabid and the period of incubation of hydrophobia is not as yet completed, and the poison of rabies is not understood to be green, a color associated in the lay mind with the most deadly and virulent toxicity.

Belief in the madstone recalls the words of Truthful James:

"Do I sleep? Do I dream?
Do I wander and doubt?
Is things what they seem,
Or is visions about?
Is our civilization a failure,
And is the Caucasian played out?"

When we have in our midst instances of fetichism and superstition that would do credit to Maoris, Veddahs and Bushmen, the conviction is warranted that our culture and enlightenment are a sham.

---

As bearing on the question of substitution and the sale of spurious drugs, the attention of our readers is called to the court decision quoted in Medical Items continued, page XIV. If drugs and medicines can be seen to be dispensed from original packages or cartons the prescriber will be better satisfied as to their genuineness.

---

Dr. T. H. Hancock has been elected president of the association of Southern Railway Surgeons. Dr. Hancock has long served the association as its efficient secretary, and the honor of election to the presidency is an acknowledgment of appreciation and esteem.

